# Testicular sex cord-stromal tumor in a boy with 2q37 deletion syndrome

**DOI:** 10.1186/1755-8794-7-19

**Published:** 2014-04-22

**Authors:** Yasunari Sakai, Ryota Souzaki, Hidetaka Yamamoto, Yuki Matsushita, Hazumu Nagata, Yoshito Ishizaki, Hiroyuki Torisu, Yoshinao Oda, Tomoaki Taguchi, Chad A Shaw, Toshiro Hara

**Affiliations:** 1 Department of Pediatrics, Graduate School of Medical Sciences, Kyushu University, Fukuoka 812-8582, Japan; 2 Department of Pediatric Surgery, Graduate School of Medical Sciences, Kyushu University, Fukuoka 812-8582, Japan; 3 Department of Pathological Sciences, Graduate School of Medical Sciences, Kyushu University, Fukuoka 812-8582, Japan; 4 Department of Molecular and Human Genetics, Baylor College of Medicine, Houston 77030, USA; 5 Department of Pediatrics, Fukuoka Dental College, Fukuoka 814-0193, Japan

**Keywords:** 2q37 deletion syndrome, Comparative genome hybridization (CGH), Copy number variation (CNV), And testicular sex cord-stromal tumor

## Abstract

**Background:**

2q37 deletion syndrome is a rare congenital disorder that is characterized by facial dysmorphism, obesity, vascular and skeletal malformations, and a variable degree of intellectual disability. To date, common but variable phenotypes, such as skeletal or digit malformations and obesity, have been associated with the deleted size or affected genes at chromosome 2q37. However, it remains elusive whether 2q37 deletion per se or other genetic factors, such as copy number variations (CNVs), may confer the risk for the tumorigenic condition.

**Case presentation:**

We report a two-year-old Japanese boy with 2q37 deletion syndrome who exhibited the typical facial appearance, coarctation of the aorta, and a global developmental delay, while lacking the symptoms of brachydactyly and obesity. He developed a sex cord-stromal tumor of the right testis at three months of age. The array comparative genome hybridization analysis identified an 8.2-Mb deletion at 2q37.1 (chr2:234,275,216-242,674,807) and it further revealed two additional CNVs: duplications at 1p36.33–p36.32 (chr1:834,101–2,567,832) and 20p12.3 (chr20:5,425,762–5,593,096). The quantitative PCRs confirmed the heterozygous deletion of *HDAC4* at 2q37.3 and duplications of *DVL1* at 1q36 and *GPCPD1* at 20p12.3.

**Conclusion:**

This study describes the unique phenotypes in a boy with 2q37 deletion and additional CNVs at 1p36.33–p36.32 and 20p12.3. The data provide evidence that the phenotypic variations and unusual complications of 2q37 deletion syndrome are not simply explained by the deleted size or genes located at 2q37, but that external CNVs may account at least in part for their variant phenotypes. Accumulating the CNV data for chromosomal disorders will be beneficial for understanding the genetic effects of concurrent CNVs on the syndromic phenotypes and rare complications.

## Background

2q37 deletion (del2q37) syndrome is a rare chromosomal disorder that is characterized by congenital hypotonia, cardiovascular anomalies, and mild to severe developmental delays [1-8]. The characteristic facial appearance includes a prominent forehead, sparse hair, highly arched eyebrows, deep-set eyes, a flat nasal bridge, a thin upper lip, and minor ear abnormalities [3]. To date, common but variable phenotypes, such as skeletal or digit malformations and obesity, have been associated with the deleted size or affected genes at 2q37 [1,3,5-7]; however, it remains unknown whether the segmental loss of 2q37 by itself or additional risk factors may contribute to the development of these phenotypes [2,3,9-13]. In this report, we describe the complication of testicular sex cord-stromal tumor as a novel complication of del2q37 syndrome. This study provides a new line of evidence for the presence of diverse genetic backgrounds in rare chromosomal disorders. This finding also supports the theory of multiple copy number variations (CNVs) as a phenotypic modifier of neuro-developmental diseases [10,14].

## Materials and methods

This study was approved by the institutional review board at Kyushu University (#461-00) and conducted in stringent compliance to the guidelines for genetic and clinical studies. Written informed consent was obtained from the parents for publication of this case report and any accompanying images. Array comparative genome hybridization (CGH) was performed as previously described [15]. Standard techniques of quantitative (q) PCR and immunohistochemistry were used [16,17]. The coordinates of CNV breakpoints were defined according to the UCSC genome assembly, GRCh37/hg19 (http://genome.ucsc.edu/). More details for these methods are described in Additional file 1.

## Case presentation

**Figure 1:**
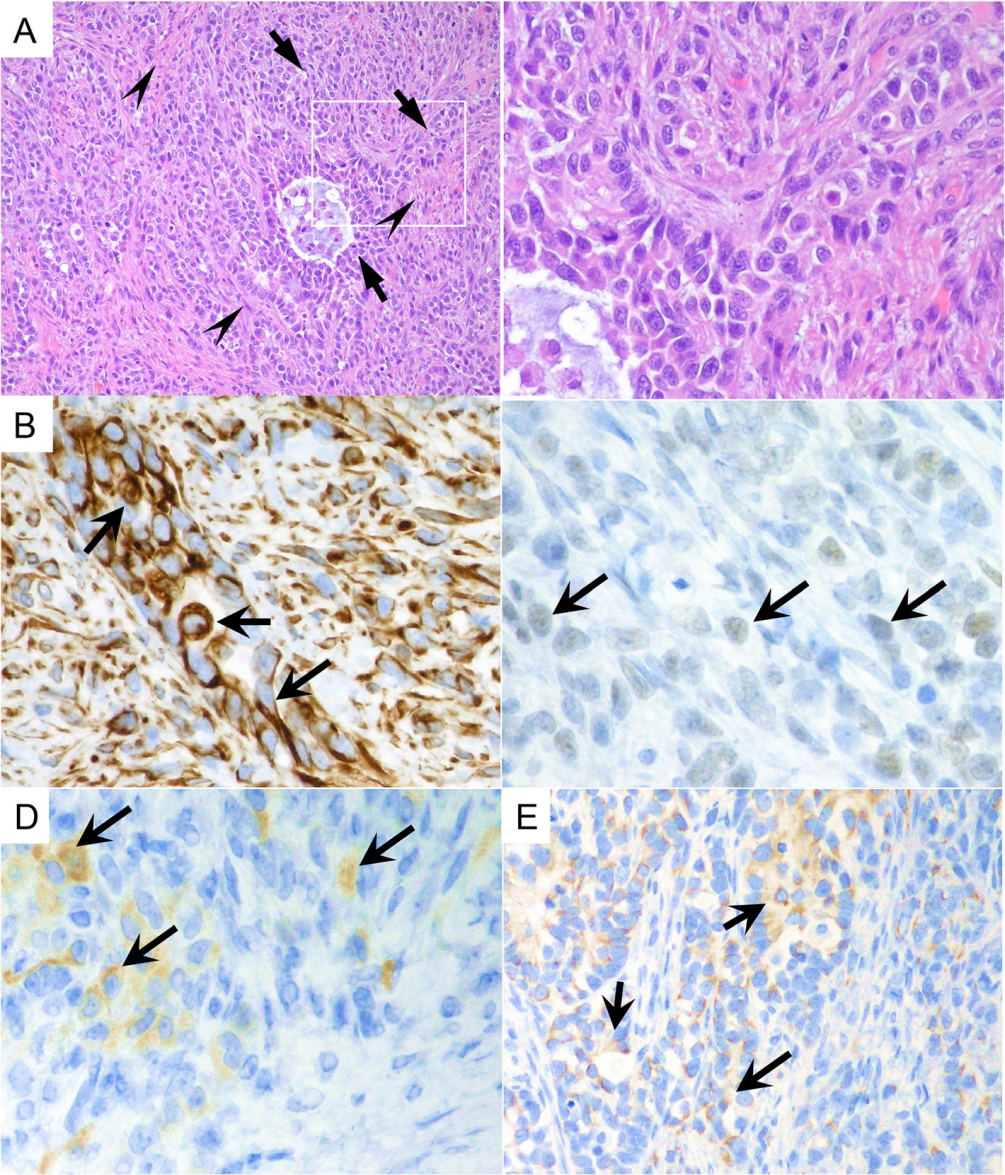
**Pathological features of the testicular tumor. (A)** The hematoxylin-eosin staining for the tumor depicts the oval or spindle-shaped tumor cells that are arranged in sheet-like or focal glandular patterns (arrows). The surrounding structures accompanying the tumor cells show fibrous bundles (arrowheads). A magnified view is shown in the right panel. **(B–E)** Immunohistochemistry for the tumor cells shows positive signals for vimentin **(B)**, alpha-inhibin **(C)**, ER **(D)**, and AE1/AE3 **(E)**. Arrows indicate the cells presenting these antigens.

A Japanese boy was born to healthy, nonconsanguineous parents at the 38th week of gestation, weighing 2,810 g, without asphyxia. Muscle hypotonia and the dysmorphic face with sparse hair, round forehead, depressed eyes, flat nasal bridge, and thin upper lips were evident (Additional file 1: Figure S1A), while brachydactyly and obesity were absent (data not shown). The G-band test determined the karyotype as 46, XY, del (2) (q37.1) (Additional file 1: Figure S1B). The karyotyping of his parents revealed that the father carried 46, XY, inv (1) (p36.1p36.3) with the breakpoint between the 1p36.1 and 1p36.3 regions (data not shown). Neither of his parents suffered from an intelligence disability or mental illness. He underwent surgical resection for right testicular hypertrophy at three months of age. Microscopic analyses disclosed the presence of a sex cord- stromal tumor (Figure 1A) and the immunohistochemical staining for vimentin, alpha-inhibin, ER, and cytokeratin AE1/AE3 supported the diagnosis (Figure 1B to E).

## Results

**Figure 2:**
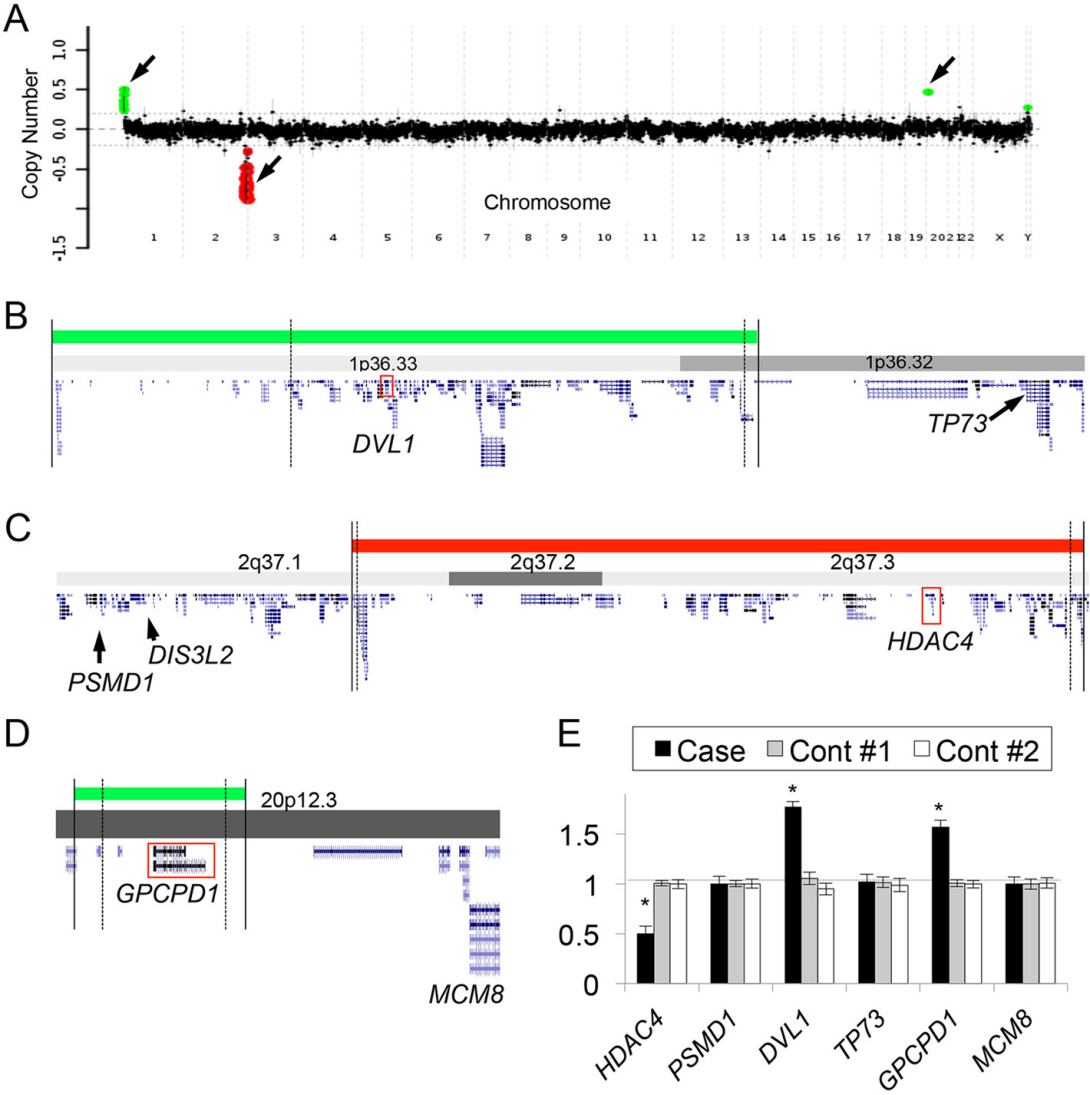
**Chromosomal microarray analysis and validation assays by quantitative PCR. (A)** A genome-wide scanning of copy number variations in the patient DNA. The plots show the relative copy numbers (Y-axis) against the chromosome regions (X-axis). Green and red dots represent the genomic regions with duplication and deletion, respectively. Arrows indicate the three chromosomal regions with CNVs (1p36, 2q37 and 20p12.3). **(B–D)** Magnified views and gene mapping in the CNV regions at 1p36.33–p36.32 **(B)**, 2q37.1–37.3 **(C)** and 20p12.3 (D). Solid and dashed vertical lines represent the maximum and minimum breakpoints of CNVs, respectively. Chromosomal bands, genomic scale and the gene loci (blue diagrams) were obtained from the UCSC genome browser (hg19). Red squares highlight the loci of the *DVL1***(B)**, *HDAC4***(C)** and *GPCPD1***(D)** genes. Only the genes that were selected for qPCR and *DIS3L2* are annotated for simplicity. **(E)** Quantitative PCR data for the copy numbers of *HDAC4*, *DVL1*, and *GPCPD1* to those of the reference genes (*PSMD1*, *TP73* and *MCM8*). Bar plots represent mean ± SD values with triplicated PCR assays. *, P < 0.05 (Student’s t-test).

The microarray-based CGH detected duplications at the chromosome 1p36.33–p36.32 and 20p12.3 regions in addition to the 8.2-Mb heterozygous deletion at 2q37 (Figure 2A). The telomeric and centromeric break points of the del2q37 were determined to be chr2:242,522,217–242,674,807 and chr2:234,275,216–234,264,038; those of dup1p36.33–p36.32 were chr1:1–834,101 and chr1:2,567,832–2,582,842; and those of dup20p12.3 were chr20:5,449,902–5,425,762 and chr20:5,593,096–5,626,442 (Figure 2B to D). We further mapped the genes within the intervals of deleted or duplicated regions according to the UCSC genome browser (Figure 2B to D). One of the Wilms tumor-associated genes, *DIS3L2* (chr2:232,826,293-233,208,678) [2], was located outside of the proximal breakpoint of our case (chr2: 234,275,216-234,264,038) (Figure 2C). Relative copy numbers of the genes within the structural variations at 2q37 (*HDAC4*), 1p36 (*DVL1*), and 20p12 (*GPCPD1*) were calculated in comparison with those of the reference genes (*PSMD1*, *TP73*, and *MCM8*) that were selected from the flanking regions of the CNVs. The qPCR analyses validated the heterozygous deletion of *HDAC4* at 2q37.3, the duplication of *DVL1* at 1p36.33, and the duplication of *GPCPD1* at 20p12.3 (Figure 2E). These data verified that an individual with del2q37 syndrome carried other genetic burdens in his genome.

**Figure 3:**
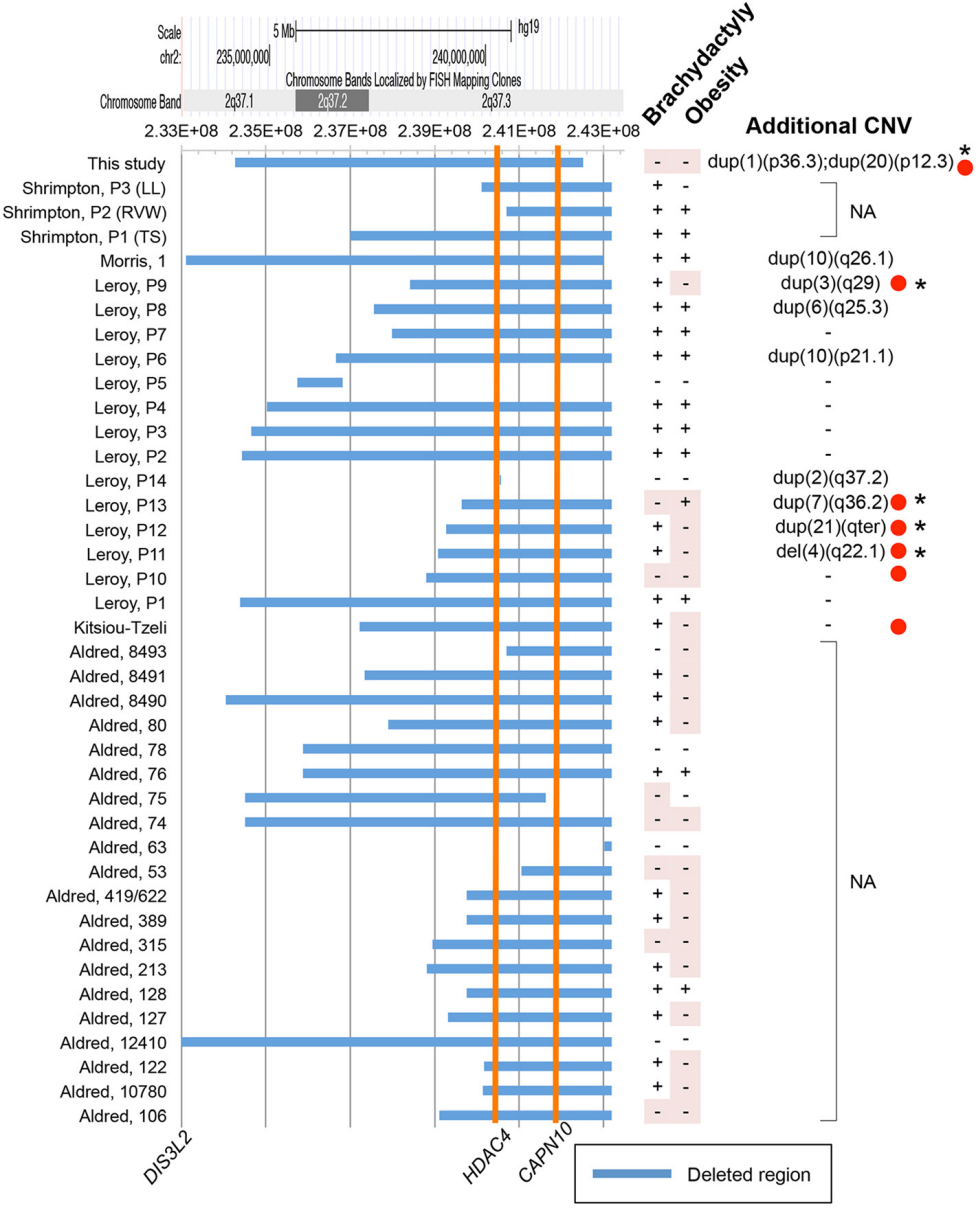
**Literature overview of the deleted regions at 2q37, the concomitant CNVs and the phenotypic variations of affected individuals.** The horizontal blue bars indicate the deleted regions that were identified in individuals with 2q37 deletion syndrome. The two orange vertical lines denote the locations of the brachydactyly- and obesity-associated genes, *HDAC4* and *CAPN10*, respectively. The information about the chromosomal bands, mapped genes, and bacterial artificial chromosome (BAC) probe loci that was described in the original studies was converted to the genomic scale of the UCSC genome browser assembly GRCh37/hg19 and is shown at the top of the bar plots. The first author and the case identification (left) are annotated as described in the original articles. The presence (+) or absence (-) of brachydactyly and obesity are shown on the right. The clinical phenotypes are highlighted (shaded in pink) when the phenotypes do not match the genotype (i.e., a patient who is negative for brachydactyly or obesity despite carrying a large deletion encompassing the *HDAC4* or *CAPN10* locus). The brief locus information for the associated CNV is described at the right end. Note that five (P9, 11, 12, 13, and this case) of the seven cases (red dots) with such atypical presentation of the phenotypes have additional CNVs that are external to 2q37. NA, data not available.

Among those with del2q37 syndrome thus far reported, we successfully identified the breakpoints at 2q37 for 39 individuals using FISH or CGH data in the literature [1,2,4-8]. To gain more insight into the genotype-phenotype correlation in individuals with del2q37 syndrome, we scrutinized the genetic information of the 39 previously reported cases from five studies, and we summarized them as a graphic overview (Figure 3) [1,2,4-8]. The size of the deletion at 2q37 ranged from 1.1 to 9.9 Mb (median, 5.2 Mb). The proximal break points greatly varied in individuals between 2q37.1 and q37.3 (chr2:232,306,614–243,026,600). Among the genes that were affected by the 2q37 deletion, the heterozygous loss of *HDAC4* and *CAPN4* have been suspected to cause brachydactyly and obesity, respectively, which are the most common clinical features of del2q37 syndrome [5]. The deleted region in our case encompassed the two gene loci, but the patient did not show either of the related phenotypes (Figure 3). Similarly, 21 out of the 40 patients (53%) with del2q37 syndrome, including our patient, showed neither brachydactyly nor obesity despite carrying large deletions encompassing the two loci (Figure 3). Furthermore, we found that five of seven cases that were atypically negative for brachydactyly and obesity carried external CNVs in addition to the 2q37 deletion (Figure 3). These data supported the idea that the size of the deletion at 2q37 and the affected genes do not always determine the phenotypic presentation of brachydactyly, obesity, and tumorigenic complications for del2q37 syndrome. Instead, some additional genetic modifiers, such as external CNVs, might contribute to the phenotypic alterations.

## Discussion

This study provides novel insight into the genotype-phenotype correlations of the rare chromosomal disorder del2q37 syndrome. First, this case manifests typical clinical features of del2q37 syndrome, while the phenotypic presentation because of the 1p36 duplication was less evident except for intelligence disability [18]. This finding suggests that haploinsufficiency of the genes at 2q37, rather than the segmental duplications of 1p36 or 20p12, shows dominant effects on the phenotypic presentation. Specific gene functions of *GPCPD1* and the duplication of 20p12 remain to be clarified, structural variations at 20p14.3, wherein this gene is located, were identified in healthy individuals of Korean ethnicity [19]. Therefore, the 20p14.3 duplication spanning the *GPCPD1* locus was unlikely to alter the phenotype of del2q37 syndrome and to produce the carcinogenic condition. While the present case did not show profound phenotype of intellectual disability compared to other cases in the literature, we focused on duplication of 1p36.33–p36.32, since the the CNVs in this region was described to confer the risk for neurodevelopmental disorders, such as autism and intellectual disability [20-22].

Recent studies supported the hypothesis that the complex phenotypes of developmental disorders might be associated with second or multiple-hit events in the genome of the affected individuals [20]. In line with this scenario, the prominent phenotype of our case might result from the combination of *HDAC4* haploinsufficiency with 1p36 duplication and/or other genetic factors that remain to be uncovered. Notably, dishevelled 1 (*DVL1*), the gene encoding the regulator of the canonical and non-canonical Wnt pathways, was located in the duplicated region of 1p36. It might be possible that the duplication of *DVL1* accelerated tumor formation in our case. Indeed, deregulated Wnt pathways and the overexpression of *DVL1* were reported in various cancers [23,24]. Altogether, we propose that the testicular tumor in our case might be a phenotypic consequence of the haploinsufficiency of *HDAC4* and the duplication of *DVL1*.

The human *HDAC4* gene encodes a chromatin remodeling factor, histone deacetylase 4, which cooperatively regulates gene expressions with other transcription factors in the physiological process of development and differentiation of various tissues [25,26]. Haploinsufficiency of *HDAC4* has been implicated as a responsible gene for the phenotypes of brachydactyly and developmental delay since the gene locus (chr2:240016312-240220334) was mapped to the deleted regions in individuals with del2q37.3 syndrome, who presented these phenotypes [8]. Moreover, Williams et al. clearly demonstrated that frame-shift mutations of *HDAC4* itself caused brachydactyly mental retardation phenotypes [8]. The deleted region in this study encompassed the *HDAC* locus, whereas he did not show the brachydactyly at two years of age. Given that brachydactyly and obesity is typically absent in early childhood of those with del2q37 syndrome, the present case may develop such phenotypes later in his life. It is thus likely that HDAC4 works as an essential regulator of gene expressions both in embryonic and postnatal development.

Drake et al. [10] studied a series of sporadic Wilms tumors and found evidence of a tumor suppressor role for a 360-kb critical region at 2q37 encompassing the *DIS3 mitotic control homolog (S. cerevisiae)-like 2* (*DIS3L2*) locus. More recently, the germline mutations within the *DIS3L2* gene were identified to cause Perlman syndrome, a congenital overgrowth syndrome that is predisposed to Wilms tumor [2]. In this report, we verified that this case had a heterozygous deletion of *HDAC4*, but not *DIS3L2* (Figures 2C and 3). *DIS3L2* was therefore unlikely to cause the testicular tumor in this case, although it cannot be completely excluded that the *DIS3L2* gene expression was deregulated in the affected tissue. Notably, elevated expression of HDAC4 is known to promote tumor formations, whereas its chemical inhibitors and siRNA-mediated knockdown of HDAC4 are associated with regressions of cell growth [27]. Therefore, it is unlikely that haploinsufficiency of *HDAC4* contributed *per se* to the testicular tumor formation in this case, whereas there is little evidence that other genes within the deleted region are associated with carcinogenesis. Concerning various unknown mechanisms underlying the unique phenotypes of this case, further genome-wide analyses to identify the unique genetic backgrounds in this case must be considered for future studies.

On the other hand, one could argue that testicular sex cord-stromal tumor is coincidental just in this case unless other cases of 2q37 deletion syndrome with similar complications are reported in the future. For better convenience in access to the clinical findings of this case, we are currently submitting the content of this report to the open resource, DECIPHER (http://decipher.sanger.ac.uk/). Also, since genome-wide data in this case are limited to the conventional CGH analysis, we must continue our efforts to identify the parental origin of accompanying CNVs, co-existence of single-nucleotide variations and their associated epigenetic as well as signaling effects [7] through genome-wide scans in future studies.

## Conclusion

In conclusion, this study raised the concept that the clinical severity and the phenotypic variety of del2q37 syndrome may not be simply associated with the size of the deletion or the genes in the affected region; rather, they might also be associated with their combination with other genetic variations, such as rare CNVs. Future studies using genome-wide scanning techniques will warrant the biological basis for phenotypic variations in affected individuals.

## Consent

Written informed consent was obtained from the parents for publication of this case report and any accompanying images. A copy of the written consent is available for review by the Editor of this journal.

## Abbreviations

Del2q37: 2q37 deletion; CGH: Comparative genome hybridization; CNV (s): Copy number variation (s).

## Competing interests

We declare that there is no potential conflict of interest for any of the authors.

## Authors’ contributions

YS and TH designed the study; YS and YM performed the genetic analyses; RS and HY carried out the immunohistochemistry; TT and YO supervised the histopathological diagnosis; YS and HY wrote the manuscript; YS and CAS organized the microarray CGH analyses; and YS, RS, YI, HT, and HN managed the patient. All authors read and approved the final manuscript.

## Pre-publication history

The pre-publication history for this paper can be accessed here:

http://www.biomedcentral.com/1755-8794/7/19/prepub

## Supplementary Material

Additional file 1: Figue S1 The face appearance and the G-band karyotype of the present case. (A) The facial appearance of the case at two years of age shows sparse hair, broad forehead, arched eyebrows, deep-set eyes with right palpebral ptosis, a flat nasal bridge, a thin upper lip (left) as well as the mild micrognathia (right), the typical features for 2q37 deletion syndrome. (B) The G-band test shows the lymphocyte karyotype of 46, XY, del (2) (q37.1). Chromosome 2 is squared with a blue line, and shown as a magnified view in the upper panel. Arrow indicates the chromosomal region with an abnormal band pattern. Click here for file
