# Supplementary material for: Testicular sex cord-stromal tumor in a boy with 2q37 deletion syndrome
Source: BMC Med Genomics. 2014 Apr 22;7:19. doi: 10.1186/1755-8794-7-19 (PMC4021669; doi:10.1186/1755-8794-7-19)
Supplement: Additional file 1: Figue S1 — The face appearance and the G-band karyotype of the present case. (A) The facial appearance of the case at two years of age shows sparse hair, broad forehead, arched eyebrows, deep-set eyes with right palpebral ptosis, a flat nasal bridge, a thin upper lip (left) as well as the mild micrognathia (right), the typical features for 2q37 deletion syndrome. (B) The G-band test shows the lymphocyte karyotype of 46, XY, del (2) (q37.1). Chromosome 2 is squared with a blue line, and shown as a magnified view in the upper panel. Arrow indicates the chromosomal region with an abnormal band pattern. [file 1755-8794-7-19-S1.pdf]

Additional Material File for

Testicular sex-cord stromal tumor in a boy with 2q37 deletion syndrome

Yasunari Sakai, Ryota Souzaki, Hidetaka Yamamoto, Yuki Matsushita, Hazumu Nagata, Yoshinao Oda, Tomoaki Taguchi, Chad A Shaw, and Toshiro Hara

This file contains the following items:

- Supplementary methods
- One supplemental Figure with its legend

There are no additional references to this information.

Supplementary Methods

PCR-based copy number assays

Quantitative (q) PCR assays were carried out using the SYBR Green system (ABI) and the relative gene dosage was calculated with the delta-delta cycle-time method as described. The forward and reverse primer sequences (5′ to 3′) for qPCR assays are as follows: aacgtgggttaaaggctgtg and agagacgcatgaggagcatt (*HDAC4*); tttttctgtgcctcccattc and ggggtttgccttactgttca (*PSMD1*); gccacacatctgtcccataa and cccacgtgtctgtgctgtag (*DVL1*); ggaggacagagatgggatga and gggcagggagaggaaaatag (*TP73*); gacggattcatccttcctga and cctggacttcctctctgtgc (*GPCPD1*); and tgtgcgctacagacaccttc and ccaagtccgcagttctcttc (*MCM8*).

Immunohistochemistry

Immunohistochemistry was performed on the formalin-fixed and paraffin-embedded specimens with primary antibodies against vimentin (clone V9, dilution 1:25; DakoCytomation, Carpinteria, CA, USA), alpha-inhibin (clone RI, dilution 1:100; Serotec, Oxford, UK), ER (clone 1D5, dilution 1:25; DakoCytomation, USA) and cytokeratin (clone AE1/AE3, dilution 1:50; Neomarkers, Fremont, CA, USA).

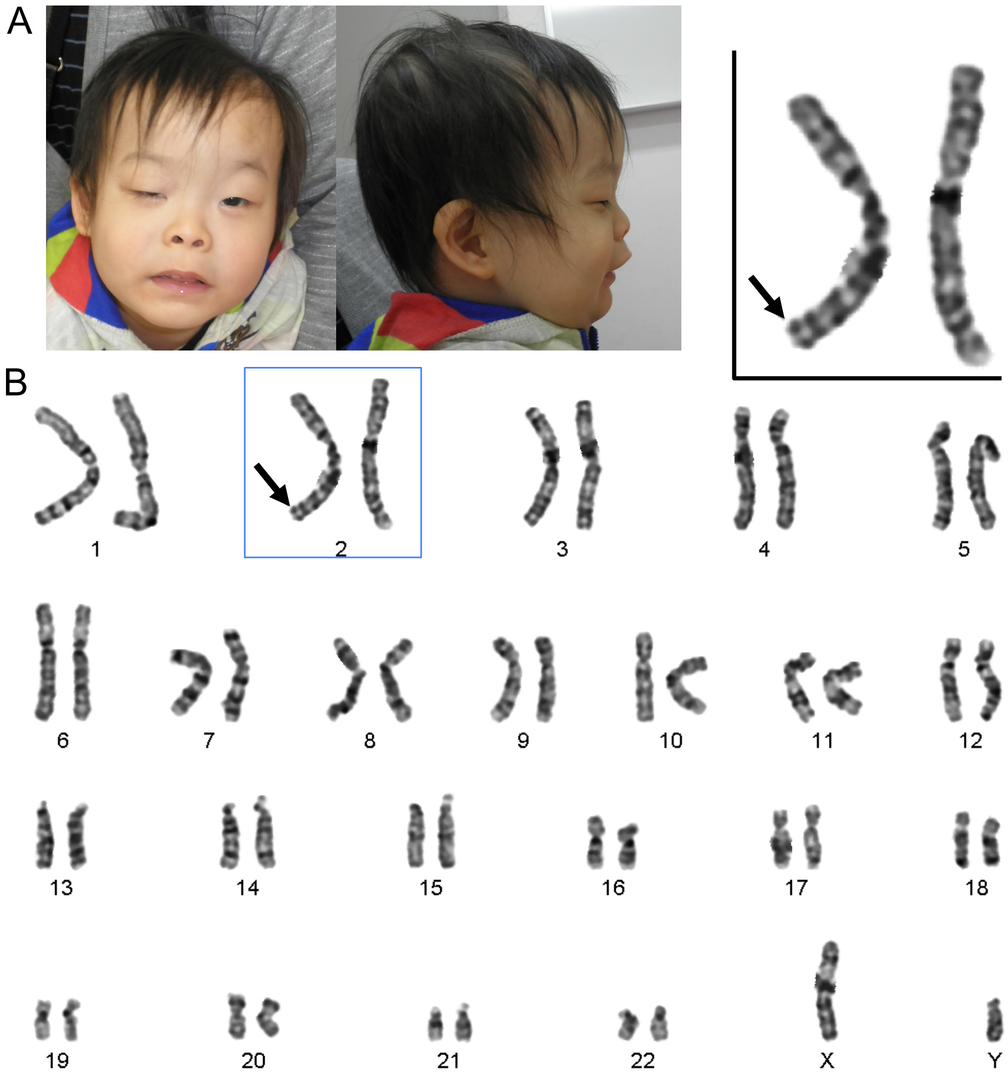

Fig S1. The face appearance and the G-band karyotype of the present case

(A) The facial appearance of the case at two years of age shows sparse hair, broad forehead, arched eyebrows, deep-set eyes with right palpebral ptosis, a flat nasal bridge, a thin upper lip (left) as well as the mild micrognathia (right), the typical features for 2q37 deletion syndrome. (B) The G-band test shows the lymphocyte karyotype of 46, XY, del(2)(q37.1). Chromosome 2 is squared with a blue line, and shown as a magnified view in the upper panel. Arrow indicates the chromosomal region with an abnormal band pattern.
